# Uncovering the characteristics of the gut microbiota in patients with ischemic stroke and hemorrhagic stroke

**DOI:** 10.1038/s41598-024-62606-x

**Published:** 2024-05-23

**Authors:** Yu-Zhu Chen, Zhao-Yong Huang, Wei-Wen Zhou, Zhong-You Li, Xiao-Peng Li, Shi-Shi Chen, Jin-Kui Ma

**Affiliations:** 1 Guangxi Zhuang Autonomous Region Center for Disease Control and Prevention, Nanning, 530028 Guangxi China; 2 School of Public Health and Management, Guangxi University of Chinese Medicine, Nanning, 530200 Guangxi Zhuang Autonomous Region China; 3 School of Food & Pharmaceutical Engineering, Zhaoqing University, Zhaoqing, 526061 Guangdong China

**Keywords:** Ischemic stroke, Hemorrhagic stroke, Intestinal microflora, Blood lipid, Genetics, Microbiology, Medical research

## Abstract

This study aimed to explore the gut microbiota characteristics of ischemic and hemorrhagic stroke patients. A case–control study was conducted, and high-throughput sequencing of the V4–V5 region of 16S rRNA was used to analyze the differences in gut microbiota. The results showed that *Proteobacteria* was significantly increased in the ischemic stroke group compared with the healthy control group, while *Fusobacteria* was significantly increased in the hemorrhagic stroke group. In the ischemic stroke group, *Butyricimonas*, *Alloprevotella*, and Escherichia were significantly more abundant than in the healthy control group. In the hemorrhagic stroke group, *Atopobium*, *Hungatella*, *Eisenbergiella*, *Butyricimonas*, *Odonbacter*, *Lachnociostridium*, *Alistipes*, *Parabacteroides*, and *Fusobacterium* were significantly more abundant than in the healthy control group. Additionally, *Alloprevotella*, *Ruminococcus*, and *Prevotella* were significantly more abundant in the ischemic stroke group than in the hemorrhagic stroke group. The gut microbiota of ischemic and hemorrhagic stroke patients has significant diversity characteristics. These results provide new theoretical basis for exploring the prevention and treatment of different types of stroke through gut microbiota research.

## Introduction

In 2019, an estimated 12.2 million people experienced a new stroke, and 101 million people were living with stroke worldwide. Stroke was responsible for 143 million disability-adjusted life years and 6.55 million deaths. Stroke remained the second leading cause of death of total deaths and the third leading cause of death and disability combined in 2019^1^. The latest Global Burden of Disease Study shows that most of the global burden of stroke originates from developing countries. China ranks the first in the world with 39.9% lifetime risk of total stroke, and the prevalence of this condition is continuously increasing^2^. There were 3.9 million new stroke cases and 1.6 million stroke deaths in China in 2020, with ischemic stroke being the most common type of stroke^3^. In Guangxi, an underdeveloped province in Southwest China, the number of inpatients with stroke has increased substantially, and the average age has become young^4^. Different stroke subtypes have various risk factors, including high systolic blood pressure, diabetes, high LDL (Low-density lipoprotein) cholesterol, smoking, low physical activity, unhealthy diet, and abdominal obesity for ischemic stroke, and hypertension for hemorrhagic stroke^1^. Recent studies have shown that intestinal microflora plays an important role in the occurrence and development of ischemic stroke^5–9^ and may participate in its pathogenesis through chronic inflammation, autonomic nervous system, and metabolism^10,11^. A cohort study showed that the intestinal microflora of patients with ischemic stroke differs from that of normal people. The abundance of *Ruminococcus* increases remarkably, whereas that of *Eubacterium* and *Bacteroides* decreases substantially in patients with ischemic stroke^12^. However, the characteristics of intestinal microflora in patients with hemorrhagic stroke are poorly reported.

Building upon recent studies that underscore the pivotal role of intestinal microflora in the genesis and progression of ischemic strokes, the current research endeavors to unravel the distinct characteristics of intestinal microflora in patients afflicted with hemorrhagic strokes. The scarcity of information in this realm calls for a comprehensive investigation to ascertain whether the microbial composition differs significantly between ischemic and hemorrhagic stroke patients. This study aims to contribute valuable insights into the potential links between intestinal microflora and stroke subtypes, paving the way for targeted interventions and therapeutic strategies tailored to the specific needs of ischemic and hemorrhagic stroke populations.

## Materials and methods

### Participants

The study was carried out between October 2016 and October 2017 in four county-level hospitals and village communities in Guangxi. Inclusion criteria for cases were established as follows: male inpatients aged 40–70 years experiencing their first stroke (in the acute phase within 5 days of onset), classified according to the diagnostic criteria outlined in the 4th cerebrovascular disease academic conference; confirmation via CT or MRI; and imaging findings consistent with clinical symptoms and signs, diagnosed as either ischemic (Group A) or hemorrhagic stroke (Group B). The healthy control group (Group C) was selected based on a 1:1 frequency match in terms of gender, age (± 4), and nationality. Healthy males in the control group were chosen from the same villages or communities where the cases were sampled.

Villages or communities were selected through simple random sampling based on the list of counties (districts) and villages or communities. A specified number of healthy individuals were then recruited, considering gender, age, and nationality. The sample composition for ischemic stroke (Group A), hemorrhagic stroke (Group B), and the healthy control group (Group C) was as follows: 7:6:13 in Wuming District, 6:5:11 in Binyang County, 2:1:3 in Gongcheng County, and 5:3:8 in Fuchuan County. Exclusion criteria for both cases and the healthy control group were implemented as follows: patients with severe liver and kidney diseases, thyroid diseases, blood diseases, autoimmune diseases; those taking anticoagulants and estrogen; and individuals who had taken antibiotics in the last 2 months or were presently suffering from intestinal diseases.

The study was approved by the Guangxi institutional review board (approval number GXIRB 2017-0005). Informed consent from all participants was obtained after full explanation of the contents was given. All participants in this study were de-identified to maintain their anonymity. All research methods in this study were performed in accordance with the approved guidelines**.**

### Instruments and reagents

The study utilized the following instruments: a precision electronic balance (Zhuojing Shanghai), oscillator vortex-5 (Kylin–Bell Haimen), electronic constant temperature stainless steel water bath (HHS-2S Shanghai), Eppendorf centrifuge (Eppendorf, Germany), electrophoresis apparatus, and gel imager (Bio Rad USA), ABI9700 PCR mete (ABI USA), Axygen Gel Extraction Kit (Axygen USA), FTC-3000TM real-time PCR (Funglyn Shanghai), HiSeq 2500 sequencer (Illumina USA), and 7600–020 automatic biochemical analyzer. Additionally, Phusion ultra-fidelity PCR Master Mix (NEB Britain), DNA marker (DL9000 Sinobio; DL2000 Takara), UltraSYBR mixture (Cwbio Beijing), HiSeq Rapid SBS Kit v2 (Illumina USA), and reagents from SiaSys Diagnostic Systems, Co., Ltd (Shanghai) were employed in the research. SRA accession number for the raw sequencing data: SRP360047:PRJNA807091, https://trace.ncbi.nlm.nih.gov/Traces/?view=study&acc=SRP360047.

### Sample and information collection

Venous blood was collected after fasting, and serum was subsequently separated. Blood fat, uric acid, high sensitivity C-reactive protein, and homocysteine levels were assessed using standardized instruments and reagents. According to a standardized sampling process and consumables, fresh fecal samples of the survey subjects are collected in dedicated fecal collection tubes in a clean environment. Among them, fecal samples from stroke cases are all collected within the first 5 days of the onset of stroke and stored at − 80 °C for further examination. Basic information and drug usage habits were obtained through a questionnaire survey.

### DNA extraction and detection of samples

Microbial DNA from the samples was extracted using the QIAamp DNA Stool Mini Kit (QIAGEN, Hilden, Germany) following the manufacturer's instructions. The integrity of the genomic DNA extracted was assessed through 1.2% agarose gel electrophoresis.

### 16S rDNA sequence amplification and MiSeq sequencing of bacteria

The V4–V5 sequence of 16S rDNA was chosen for high-throughput sequencing analysis. A two-step PCR approach was employed to construct the library. Purified DNA served as the template for PCR amplification, utilizing the 16S rDNA V4-V5 universal primer [515F (5ʹ-GTGCCAGCMGCCGCGG-3ʹ) and 926R (5ʹ-CCGTCAATTCMTTTGAGTTT-3ʹ)], along with a fusion primer containing some Hiseq sequencing primer and barcode sequence for detection via 1.2% agarose gel electrophoresis. Samples exhibiting favorable detection results underwent recovery through 2% agarose gel electrophoresis. Subsequently, eight cycles of PCR amplification were conducted using the recovered product as the template. The primer employed was a fusion primer containing a hiseq connector, barcode, and sequencing primer. AxyPrepDNA Gel Extraction Kit (AXYGEN company) was used for the recovery of all PCR products. Fluorescence quantification was achieved using an FTC-3000TM Real-Time PCR meter. Following homogenization and mixing, the library was constructed. Illumina Hiseq PE250 sequencing was carried out utilizing HiSeq Rapid SBS Kit v2 at TinyGene Biotechnical, Co., Ltd. (Shanghai).

The first PCR reaction system consisted of the following components: 10 μL of 5 × buffer, 1 μL of dNTP (10 mM), 1U of Phusion ultra-fidelity DNA polymerase, 1 μL each of forward and reverse primers (10 mM), and 20–50 ng of template DNA, with ultrapure water added to reach a total volume of 50 μL. The PCR reaction conditions were as follows: an initial denaturation at 94 °C for 2 min, followed by 25 cycles of denaturation at 94 °C for 30 s, annealing at 56 °C for 30 s, extension at 72 °C for 30 s, and a final extension at 72 °C for 5 min.

In the second PCR reaction system, the components included 8 μL of 5 × buffer, 1 μL of dNTP (10 mM), 0.8 U of Phusion ultra-fidelity DNA polymerase, 1 μL each of forward and reverse primers (10 mM), and 5 μL of template DNA, with ultrapure water added to achieve a total volume of 40 μL. The PCR reaction conditions were as follows: an initial denaturation at 94 °C for 2 min, followed by eight cycles of denaturation at 94 °C for 30 s, annealing at 56 °C for 30 s, extension at 72 °C for 30 s, and a final extension at 72 °C for 5 min, with the temperature maintained at 10 °C during the last step.

### Data analysis

Based on the original data, sample reads were distributed by barcode, and the valid sequence for each sample was obtained. Trimmomatic software (version 0.35, http://www.usadellab.org/cms/?page=trimmomatic) was employed to eliminate low-quality sequences at the end of the sequencing results. Considering the overlap relationship between paired-end (PE) reads, Flash software (version 1.2.11, https://ccb.jhu.edu/software/FLASH/) was utilized to concatenate read pairs into a single sequence. Mothur software (version 1.33.3, https://mothur.org/) was employed to control and filter sequence quality, removing ambiguous, homologous, excessively long, and short sequences, as well as certain chimeras generated during the PCR process, to acquire optimal sequences. Operational Taxonomic Unit (OTU) UPARSE software (usearch version v8.1.1756, https://drive5.com/usearch/manual8.1/uparse_pipeline.html) was applied, and representative sequences were compared with the Silva 128 database for species information annotation. The community structure was statistically analyzed at the classification levels of phylum, class, order, family, genus, and species.

A sequence of statistical analyses and visual mapping of community structure and phylogeny (unifrac) was carried out. Mothur software (version 1.33.3, https://mothur.org/) was utilized for Alpha diversity analysis, encompassing Chao, Ace, and other species richness statistics, as well as Shannon, Simpson, and other species diversity statistics. Additionally, VENN map, dilution curve, Beta diversity analysis (un), and weighted UniFrac analysis were conducted using Mothur software (version 1.33.3, https://mothur.org/). R language (version 3.6.3, https://www.r-project.org/) was employed for beta diversity analysis based on species. The Kruskal–Wallis test was employed to compare the three groups, while the Wilcoxon signed-rank test was applied for comparing two groups of samples. Heatmap mapping, multi-sample similarity tree drawing, and PCA analysis were also undertaken. The non-parametric factorial Kruskal–Wallis (KW) sum-rank test was utilized to detect significant differences in abundance and identify related groups. Finally, LEfSe was employed to estimate the impact of each component's (species) abundance on the difference effect based on linear discriminant analysis (LDA).

For blood biochemical examination, SPSS 21.0 was used. Comparison among three groups was conducted using ANOVA or multi-sample nonparametric tests, and comparison between two groups was performed using t-tests. The nonparametric test was adopted under unequal variance and non-normal distribution.

## Results

Seventy males were enrolled in this research. Among them, 20 experienced ischemic stroke, 15 had hemorrhagic stroke, and 35 were deemed healthy. The patients with ischemic stroke ranged in age from 43 to 69 years, with an average age of 57.23 ± 8.61 years. Those with hemorrhagic stroke were aged between 43 and 70 years, with an average age of 61.07 ± 8.79 years. The healthy control group had individuals aged between 43 and 70 years, with an average age of 59.41 ± 8.66 years. No statistically significant difference in age was identified among the three groups (F = 1.029, *P* = 0.363). Furthermore, no statistically significant differences were observed in age, physical activity, smoking, drinking, height, BMI, total cholesterol, triglyceride, HDL, LDL, high sensitivity C-reactive protein, uric acid, homocysteine, energy, protein, and fat intake among the three groups (*P* > 0.05) (Table 1).

**Table 1:** Comparison of biological indexes among patients with ischemic and hemorrhagic strokes and healthy control ( ± s).

Index	Ischemic stroke (n = 20)	Hemorrhagic stroke (n = 15)	Healthy control (n = 35)	*P*
Age (y)	57.23 ± 8.61	61.07 ± 8.79	59.41 ± 8.66	0.363
Height (cm)	163.74 ± 5.12	166.69 ± 4.99	162.94 ± 6.62	0.159
Weight (kg)	62.16 ± 8.83	64.85 ± 10.06	61.46 ± 10.05	0.565
BMI (kg/m^2^)	23.18 ± 3.09	23.32 ± 3.41	23.07 ± 2.98	0.968
TC (mmol/L)	4. 65 ± 1.58	5.93 ± 1.54	4.97 ± 1.98	0.601
TG (mmol/L)	1.56 ± 0.86	1.70 ± 0.72	1.63 ± 1.27	0.931
HDL (mmol/L)	1.23 ± 0.44	1.46 ± 0.68	1.36 ± 0.65	0.537
LDL (mmol/L)	2.72 ± 1.11	3.70 ± 1.28	2.66 ± 2.10	0.171
UA (μmol/L)	315.00 ± 115.14	282.00 ± 228.87	327.09 ± 148.79	0.687
Hs C-creactive protein (mg/L)	9.18 ± 18.94	12.55 ± 19.64	3.96 ± 4.96	0.285
Homocysteine (μmol/L)	20.88 ± 18.93	22.92 ± 9.21	20.66 ± 5.64	0.601
Energy (kcal)	1916.45 ± 472.08	2162.26 ± 928.57	1763.10 ± 496.06	0.127
Protein (g)	64.73 ± 25.26	83.11 ± 51.17	57.44 ± 25.13	0.054
% of energy intake	13.18 ± 2.24	14.48 ± 2.47	12.68 ± 2.00	0.045
Fat (g)	80.63 ± 28.97	100.83 ± 58.76	70.13 ± 23.21	0.077
% of energy intake	37.46 ± 7.48	40.38 ± 5.92	35.73 ± 5.65	0.081
Carbohydrate (g)	232.98 ± 62.35	230.58 ± 59.93	225.55 ± 62.39	0.909
% of energy intake	49.35 ± 9.50	45.14 ± 7.94	51.59 ± 7.22	0.056
Physical activity				
Light	8 (35.0%)	6 (38.5%)	16 (35.0%)	0.814
Medium	4 (35.0%)	3 (23.1%)	5 (35.0%)	
Heavy	8 (30.0%)	6 (38.5%)	14 (30.0%)	
Do you smoke now				
No	7 (35.0%)	7 (46.2%)	17 (47.1%)	0.760
Yes	13 (65.0%)	8 (53.8%)	18 (52.9%)	
Do you drink now				
No	7 (31.6%)	4 (23.1%)	12 (35.3%)	0.723
Yes	13 (68.4%)	11 (76.9%)	23 (64.7%)	

### Sequencing data

A sum of 4,627,827 valid sequences was identified among the 70 samples from the three groups. On average, each sample contained 66,069 sequences (range: 54,134–68,854). A total of 3,813,520 optimal sequences were obtained, and their average length fell within the range of 400–500 bp. Clustering with 97% similarity resulted in a total of 535 Operational Taxonomic Units (OTUs). Results showed that the ischemic stroke group (A) yielded 615 OTUs, the hemorrhagic stroke group (B) produced 543 OTUs, and the healthy control group (C) had 606 OTUs. Notably, a total of 477 OTUs were identified across all three groups.

### Alpha diversity analysis

**Figure 1:**
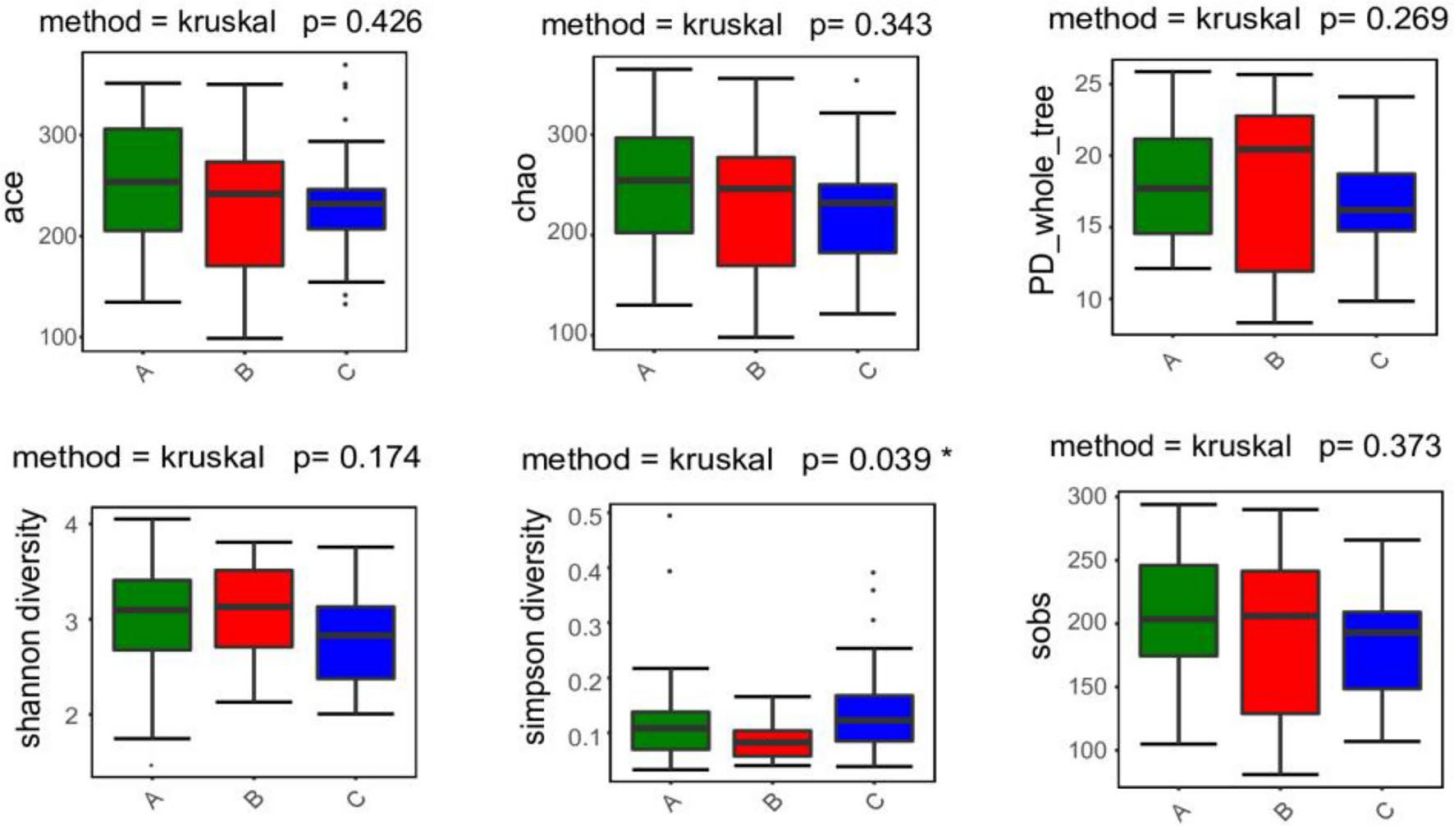
The results of alpha diversity analysis for the ischemic stroke group (A), hemorrhagic stroke group (B), and healthy control group (C).

Statistically significant differences were found in the Shannon index (*P* < 0.01), but not in the Ace, Chao, PD whole tree, Shannon, and Sobs indices (*P* > 0.05) among the three groups (Fig. 1).

### Structural analysis of bacterial colony

**Figure 2:**
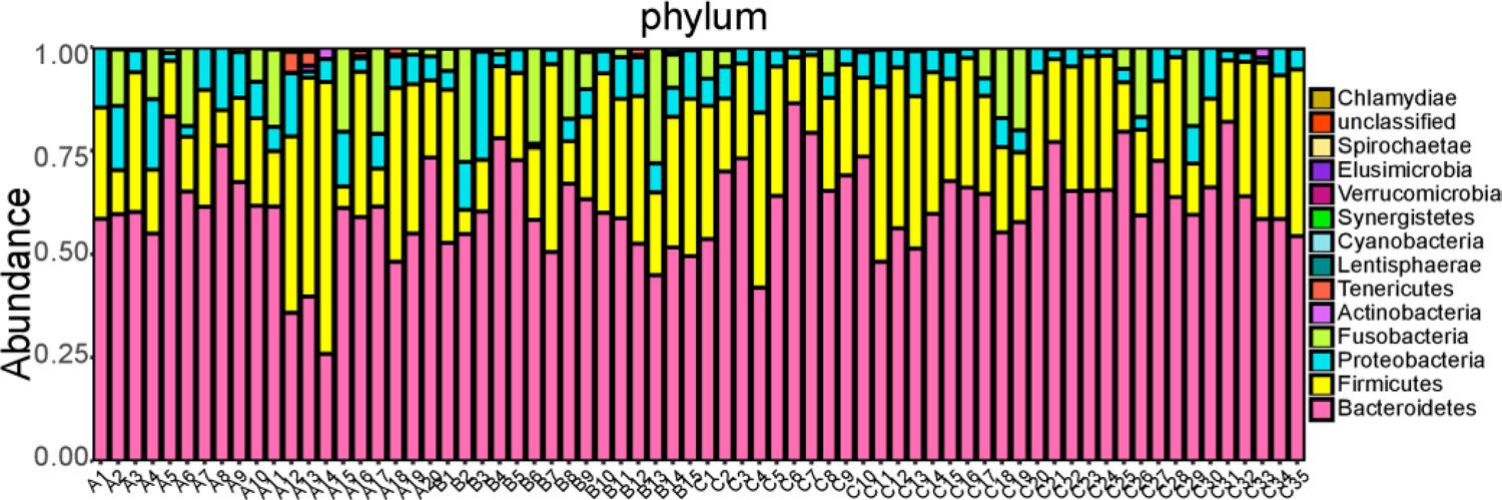
Bar charts of the distribution of intestinal microflora in people with ischemic stroke (A), hemorrhagic stroke (B), and the healthy control group (C) at the phylum level. Note: A1 ~ A20 are samples of the intestinal microflora of people with ischemic stroke; B1 ~ B15 are samples of the intestinal microflora of people with hemorrhagic stroke; C1 ~ C35 are samples of the intestinal microflora of people in the healthy control group.

**Figure 3:**
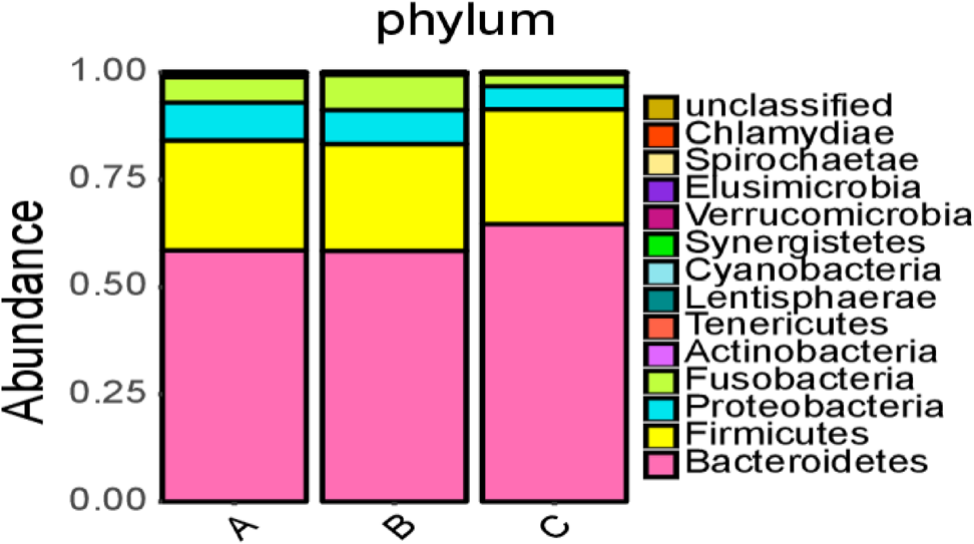
Composition of intestinal microflora in people with ischemic stroke (A), hemorrhagic stroke (B), and the healthy control group (C) at the phylum level.

At the phylum level, 14 floras were identified across the three groups: *Bacteroidetes*, *Firmicutes*, *Proteobacteria*, *Fusobacteria*, *Tenericutes*, *Actinobacteria*, *Lentisphaerae*, *Verrucomicrobia*, *Cyanobacteria*, Unclassified, *Synergistetes*, *Spirochaetae*, *Elusimicrobia*, and *Chlamydiae*. The dominant phyla in all samples were *Bacteroidetes*, *Firmicutes*, *Proteobacteria*, *Fusobacteria*, *Actinobacteria*, and *Tenericutes*, constituting 58.53%, 25.60%, 8.77%, 5.99%, 0.33%, and 0.58%, respectively, in the ischemic stroke group; 58.37%, 24.95%, 7.88%, 8.24%, 0.24%, and 0.12%, respectively, in the hemorrhagic stroke group; and 64.63%, 26.73%, 5.33%, 3.02%, 0.17%, and 0.03%, respectively, in the healthy control (Fig. 3). The Kruskal–Wallis H test was employed to analyze significant differences in species abundance among the three groups. The results indicated statistical significance in the distribution of *Proteobacteria* and *Fusobacteria* among the three groups (*P* < 0.05), as shown in Figs. 2 and 3.

**Figure 4:**
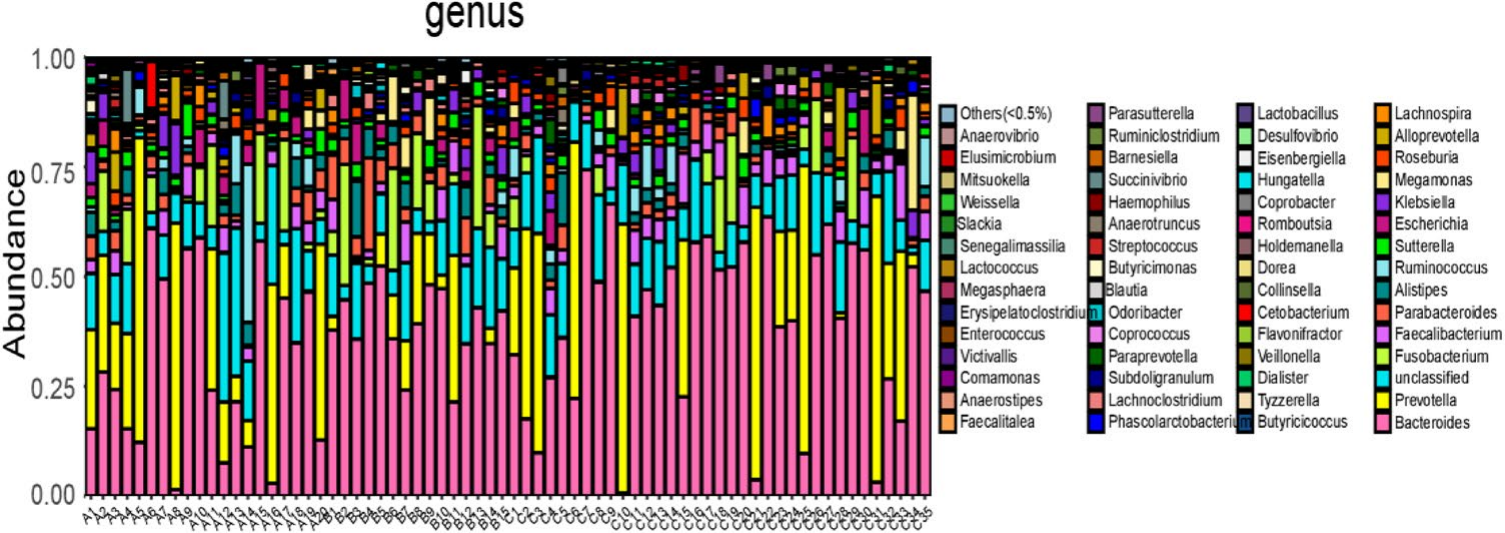
Bar charts of the distribution of intestinal microflora in people with ischemic stroke (A), hemorrhagic stroke (B), and the healthy control group (C) at the genus level. Note: A1 ~ A20 are samples of the intestinal microflora of people with ischemic stroke; B1 ~ B15 are samples of the intestinal microflora of people with hemorrhagic stroke; C1 ~ C35 are samples of the intestinal microflora of people in the healthy control group.

**Figure 5:**
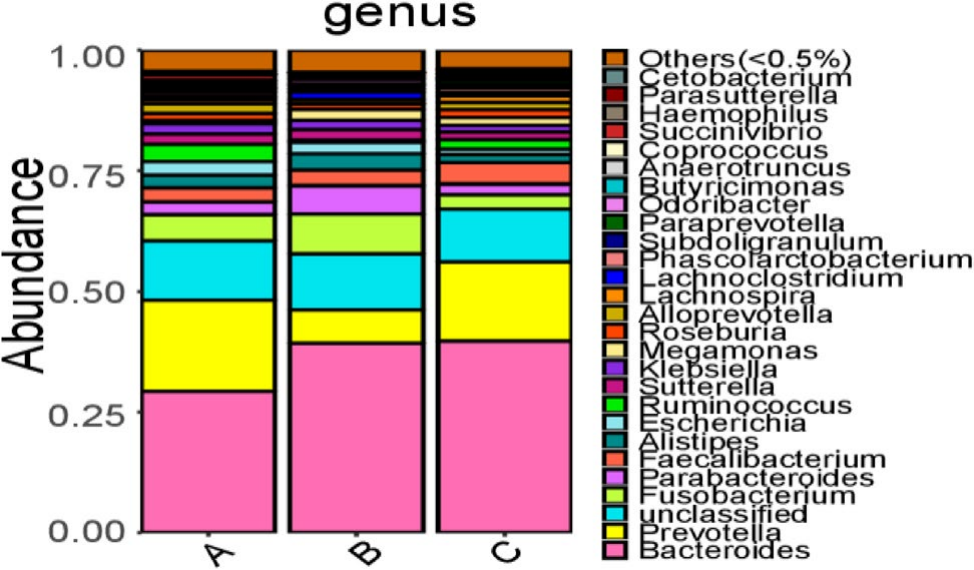
Composition of intestinal microflora in people with ischemic stroke (A), hemorrhagic stroke (B), and the healthy control group (C) at the genus level.

At the genus level, a total of 154 genera of floras were identified in all samples. Among them, 13 were abundant (constituting more than 1.0%) in the ischemic stroke group and were ranked from high to low abundance as follows: *Bacteroides* 29.35%, *Prevotella* 18.86%, *Fusobacterium* 5.46%, *Ruminococcus* 3.62%, *Faecalibacterium* 2.87%, *Escherichia* 2.75%, *Parabacteroides* 2.63%, *Alistipes* 2.57%, *Klebsiella* 2.15%, *Sutterella* 2.13%, *Alloprevotella* 1.96%, *Roseburia* 1.47%, and *Succinivibrio* 1.06%. In the hemorrhagic stroke group, twelve genera were abundant (accounting for more than 1.0%) and were ranked from high to low abundance as follows: *Bacteroides* 39.31%, *Fusobacterium* 8.24%, *Prevotella* 6.94%, *Parabacteroides* 5.83%, *Alistipes* 3.38%, *Faecalibacterium* 3.19%, *Escherichia* 2.39%, *Megamonas* 2.23%, *Sutterella* 2.23%, *Klebsiella* 1.90%, *Lachnoclostridium* 1.50%, and *Roseburia* 1.12%. In the healthy control group, fifteen genera were abundant (constituting more than 1.0%) and were ranked from high to low abundance as follows: *Bacteroides* 39.78%, *Prevotella* 16.41%, *Faecalibacterium* 4.38%, *Fusobacterium* 3.02%, *Parabacteroides* 2.17%, *Ruminococcus* 2.00%, *Alistipes* 1.65%, *Megamonas* 1.64%, *Roseburia* 1.62%, *Sutterella* 1.59%, *Alloprevotella* 1.51%, *Klebsiella* 1.35%, *Lachnospira* 1.32%, and *Escherichia* 1.12% (Figs. 4, 5).

### Comparative analysis on differences in intestinal microflora among the ischemic stroke group, hemorrhagic stroke group, and healthy control group

**Figure 6:**
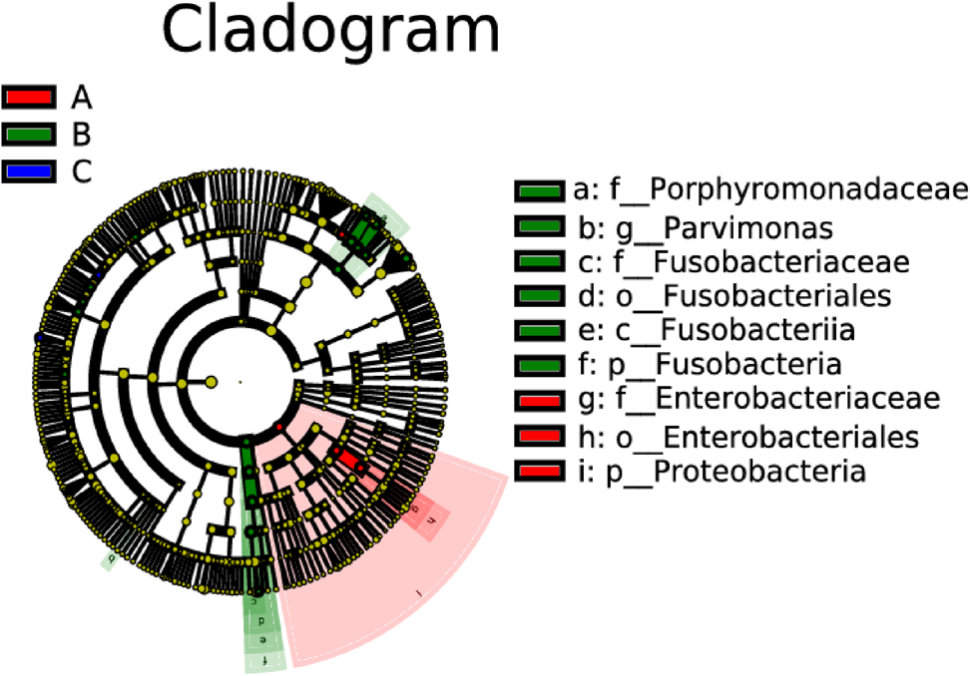
Lefse analysis of intestinal microflora in people with ischemic stroke (A), hemorrhagic stroke (B), and the healthy control group (C). Note: The classification tree, from the inner ring to the outer ring, displayed the subordination of species in turn. The node size corresponded to the average relative abundance of species. Yellow nodes indicated insignificant differences among functional groups, while red or green regions indicated high differences in species abundance for each group.

**Figure 7:**
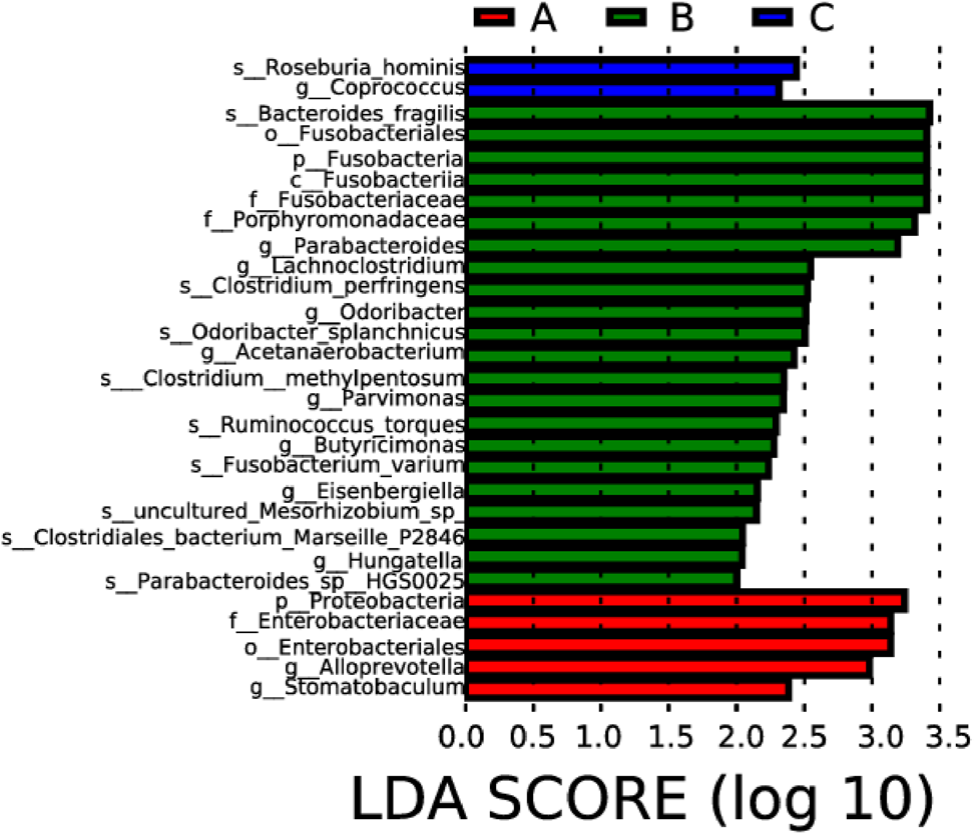
LDA bar charts of intestinal microflora in people with ischemic stroke (A), hemorrhagic stroke (B), and the healthy control group (C). Note: In LDA scoring among different groups, the X-axis represents LDA SCORE (log), and the Y-axis represents significantly different bacterial categories in stool (LDA SCORE > 2).

Differences in various taxonomic levels (phylum, class, order, family, genus, and species) among the three groups of intestinal microflora were analyzed. The results indicated that *Proteobacteria*, *Alloprevotella*, and *Stomatobaculum* were significantly higher in the ischemic stroke group compared to the other two groups. *Fusobacteria*, *Hungatella*, *Eisenbergiella*, *Butyricimonas*, *Parvimonas*, *Acetanaerobacterium*, *Odoribacter*, *Lachnoclostridium*, and *Parabacteroides* were significantly higher in the hemorrhagic stroke group compared to the other two groups. *Coprococcus* and *Roseburia hominis* were significantly higher in the healthy control group than in the other two groups (Figs. 6, 7).

### Comparative analysis on differences in intestinal microflora between ischemic stroke group and healthy control group

**Figure 8:**
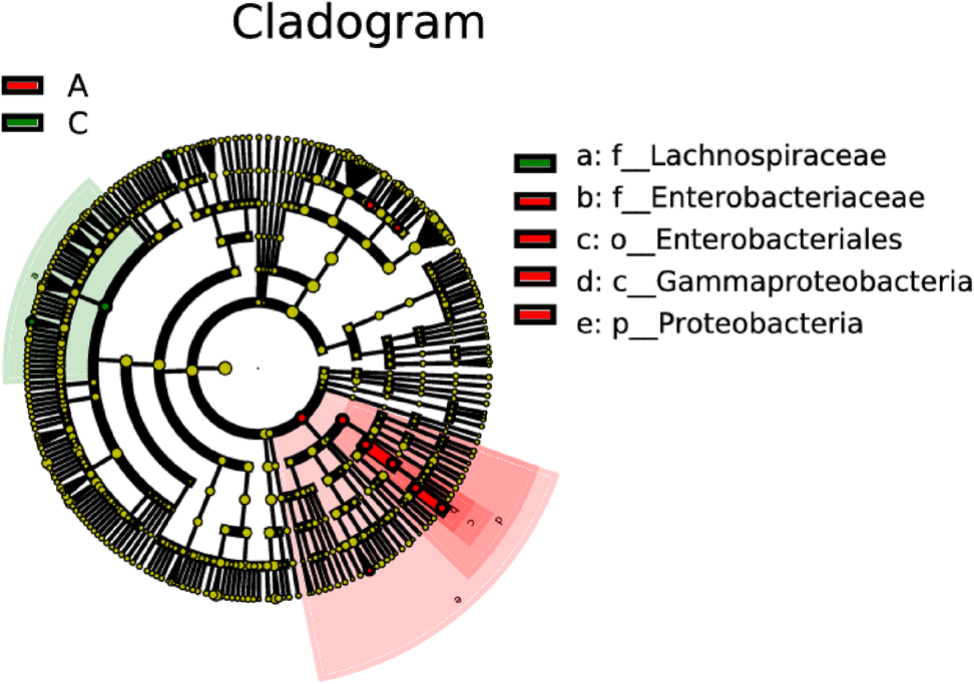
Lefse analysis on intestinal microflora between the ischemic stroke group (A) and the healthy control group (C). Note: The classification tree, from the inner ring to the outer ring, displayed the subordination of species in turn. The node size corresponded to the average relative abundance of species. Yellow nodes indicated insignificant differences among functional groups, while red or green regions indicated high differences in species abundance for each group.

**Figure 9:**
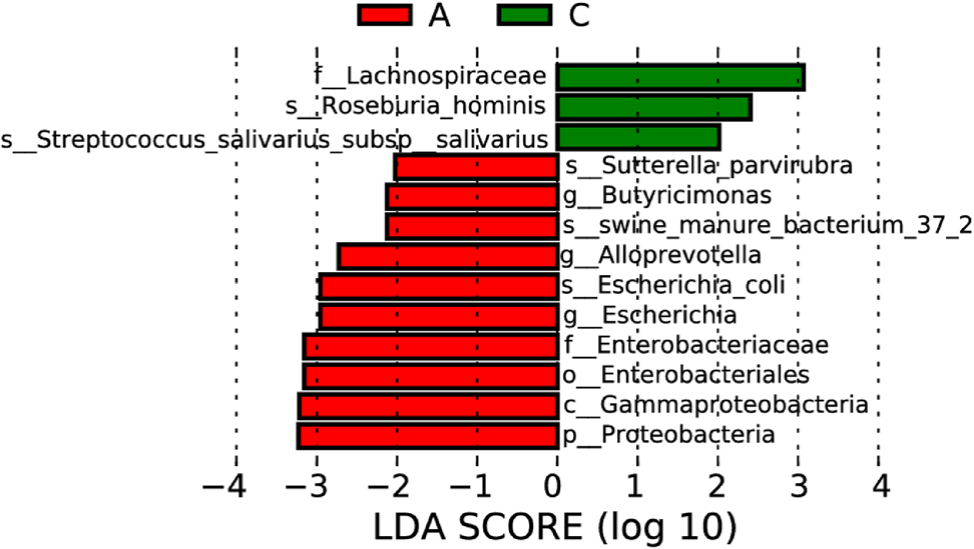
LDA on intestinal microflora between the ischemic stroke group (A) and the healthy control group (C). Note: In LDA scoring among different groups, the X-axis represents LDA SCORE (log), and the Y-axis represents significantly different bacterial categories in stool (LDA SCORE > 2).

In this comparative analysis of intestinal microflora between the ischemic stroke group and the healthy control group, linear discriminant analysis was conducted based on two independent samples. Distinctive microbial patterns associated with ischemic stroke were sought through this analysis. The outcomes of this analysis revealed intriguing variations in microbial abundances between the ischemic stroke and healthy control groups. Specifically, the abundances of *Butyricimonas*, *Alloprevotella*, and *Escherichia* were found to be significantly higher in the ischemic stroke group compared to the healthy control, as illustrated in Figs. 8 and 9. Potential associations between these specific microbial taxa and the pathophysiological mechanisms underlying ischemic stroke are suggested, urging further investigation into their roles in stroke-related outcomes.

Conversely, a noteworthy finding emerged as the abundances of *Roseburia* and *Streptococcus salivarius* were significantly higher in the healthy control group as opposed to the ischemic stroke group. This differential microbial profile raises questions about the potential protective or regulatory roles these genera might play in the context of ischemic stroke, thereby warranting in-depth exploration of their implications for cerebrovascular health.

### Comparative analysis on differences in intestinal microflora between hemorrhagic stroke group and healthy control group

**Figure 10:**
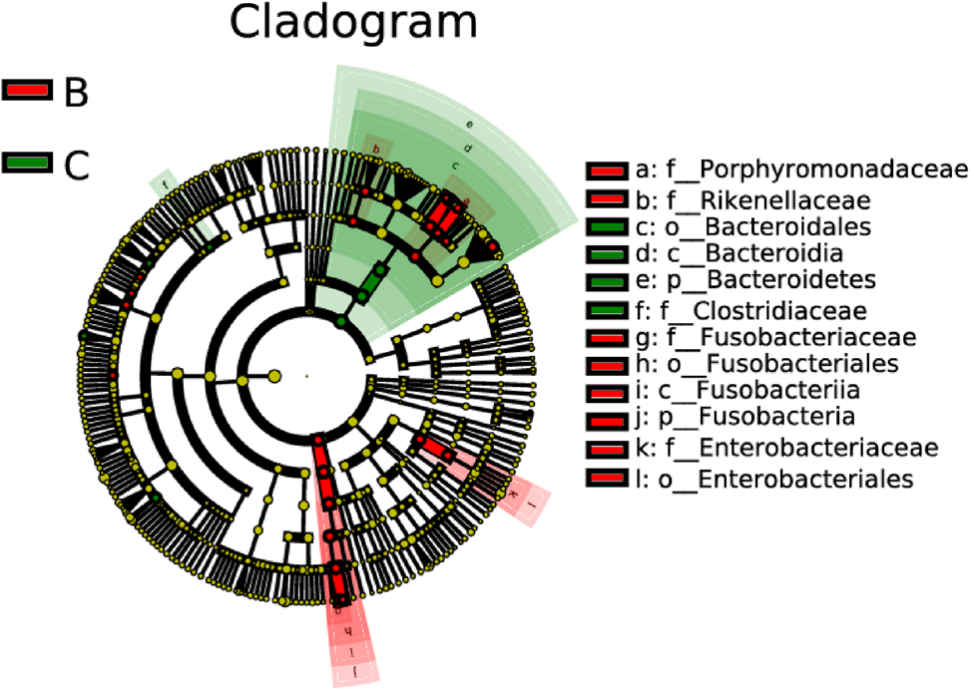
Lefse analysis on intestinal microflora between the hemorrhagic stroke group (B) and the healthy control group (C). Note: The classification tree, from the inner ring to the outer ring, displayed the subordination of species in turn. The node size corresponded to the average relative abundance of species. Yellow nodes indicated insignificant differences among functional groups, while red or green regions indicated high differences in species abundance for each group.

**Figure 11:**
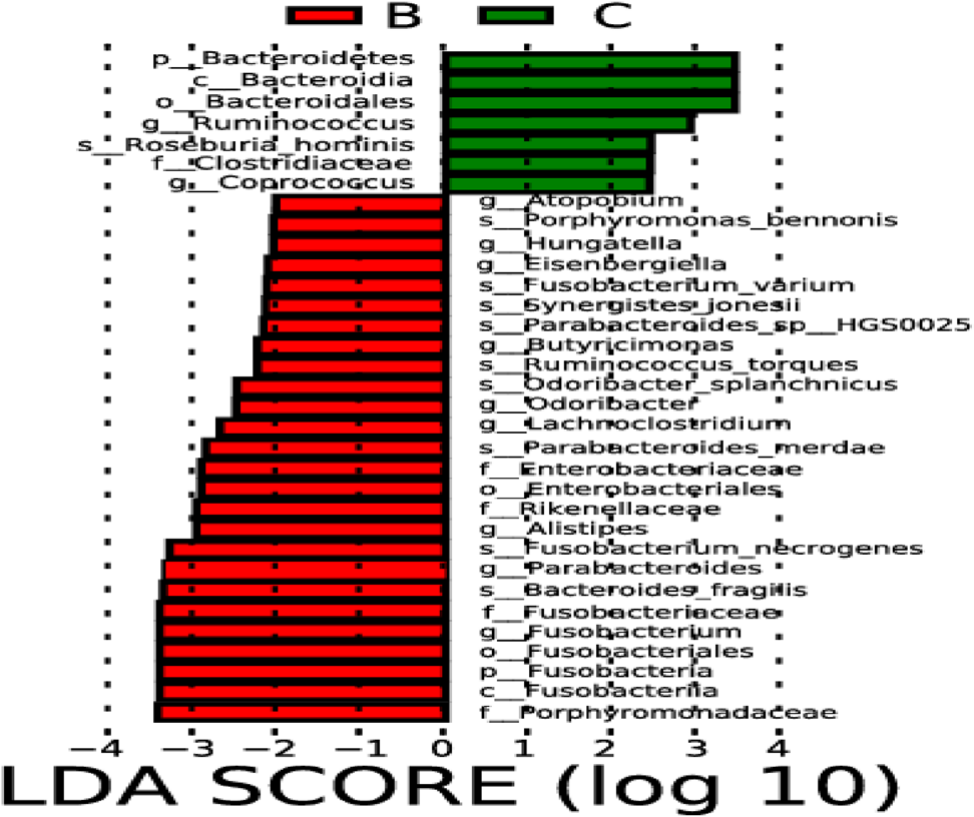
LDA on intestinal microflora between the hemorrhagic stroke group (B) and the healthy control group (C). Note: In LDA scoring among different groups, the X-axis represents LDA SCORE (log), and the Y-axis represents significantly different bacterial categories in stool (LDA SCORE > 2).

In this comparative analysis of differences in intestinal microflora between the hemorrhagic stroke group and the healthy control group, notable distinctions emerged in the abundances of specific genera. The hemorrhagic stroke group exhibited significantly higher levels of *Atopobium*, *Hungatella*, *Eisenbergiella*, *Butyricimonas*, *Odoribacter*, *Lachnoclostridium*, *Alistipes*, *Parabacteroides*, and *Fusobacterium* when contrasted with the healthy control group, as depicted in Figs. 10 and 11. This elevation in certain microbial taxa suggests a potential association between these genera and the pathophysiological mechanisms underlying hemorrhagic stroke.

Conversely, intriguingly, the abundances of *Ruminococcus* and *Coprococcus* were found to be significantly higher in the healthy control group in comparison to the hemorrhagic stroke group. This observation prompts further exploration into the potential protective role of these particular genera in maintaining intestinal microbial balance and their probable implications for overall health.

### Comparative analysis on differences in intestinal microflora between ischemic stroke group and hemorrhagic stroke group

**Figure 12:**
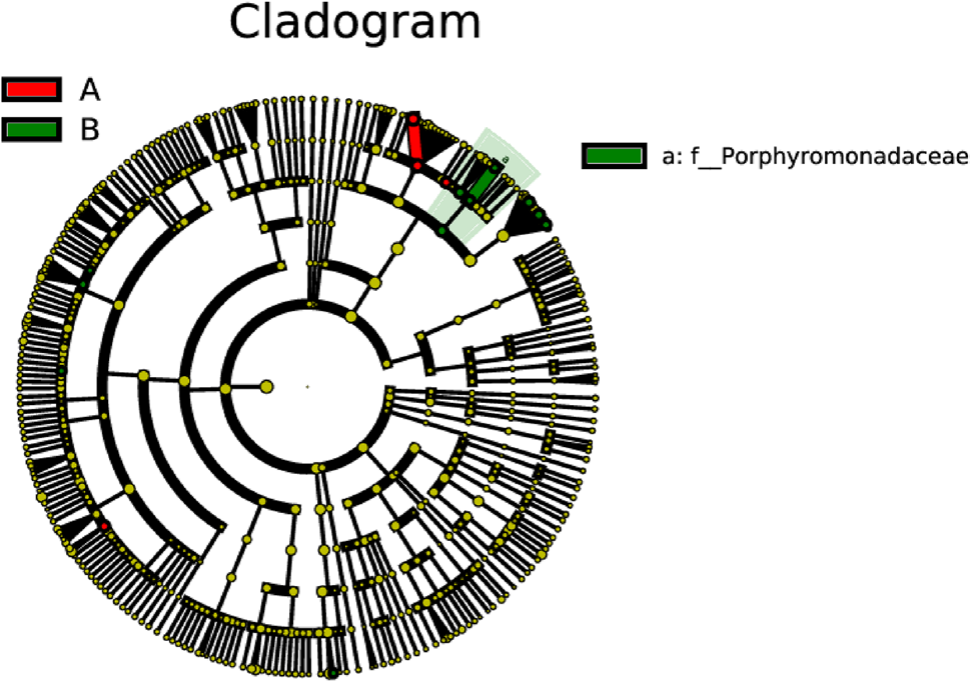
Lefse analysis on intestinal microflora between the ischemic stroke group (A) and the hemorrhagic stroke group (B). Note: The classification tree, from the inner ring to the outer ring, displayed the subordination of species in turn. The node size corresponded to the average relative abundance of species. Yellow nodes indicated insignificant differences among functional groups, while red or green regions indicated high differences in species abundance for each group.

**Figure 13:**
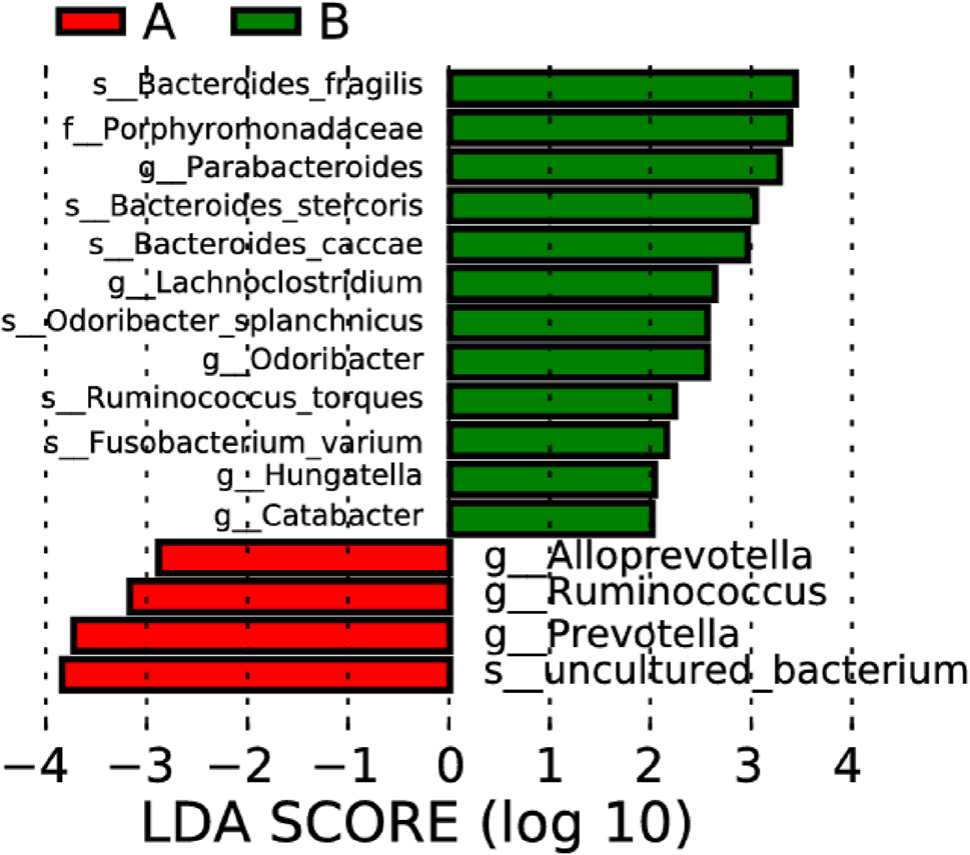
LDA on intestinal microflora between the ischemic stroke group (A) and the hemorrhagic stroke group (B). Note: In LDA scoring among different groups, the X-axis represents LDA SCORE (log), and the Y-axis represents significantly different bacterial categories in stool (LDA SCORE > 2).

In the comparative analysis on differences in intestinal microflora between the ischemic stroke group and the hemorrhagic stroke group, notable distinctions were identified. The abundances of *Alloprevotella*, *Ruminococcus*, and *Prevotella* were observed to be significantly higher in the ischemic stroke group when compared to the hemorrhagic stroke group. Conversely, the abundances of *Parabacteriodes*, *Lachnoclostridium*, *Odoribacter*, *Hungatella*, and *Catabacter* exhibited a significant increase in the hemorrhagic stroke group in comparison to the ischemic stroke group (as depicted in Figs. 12 and 13). These findings suggest a distinct microbial composition in the gut microbiota between individuals with ischemic and hemorrhagic strokes, emphasizing the potential impact of stroke subtype on the intestinal microbial landscape.

## Discussion

The bacterial phyla observed in all samples constituted *Bacteroidetes*, *Firmicutes*, *Proteobacteria*, *Fusobacteria*, *Actinobacteria*, and *Tenericutes*, collectively accounting for more than 98% of the total. A statistically significant overall distribution of *Proteobacteria* and *Fusobacteria* was identified among the three groups. Specifically, there was a significant increase in the abundance of *Proteobacteria* in ischemic stroke, while the abundance of *Fusobacteria* showed a notable increase in hemorrhagic stroke. The abundance of *Fusobacterium* within the *Fusobacteria* category was elevated in both ischemic and hemorrhagic stroke groups compared to the healthy control, with a particularly significant increase observed in the hemorrhagic stroke group. These findings align with a prior study by Yin et al.^13^, which reported a similar trend of increased *Proteobacteria* and decreased *Bacteroidetes* in ischemic stroke. Furthermore, the positive correlation between the abundances of *Proteobacteria* and *Fusobacteria* with the occurrence of intestinal inflammation, as highlighted by Wiredu Ocansey, Hang et al.^14^, underscores the potential involvement of these bacterial phyla in inflammatory processes. *Fusobacterium*, as indicated by studies conducted by Wu et al.^15^ and Fan et al.^16^, emerges as a significant player linked to inflammatory bowel disease, exhibiting functions of invasion, adhesion, and proinflammation. In summary, the correlations observed between *Proteobacteria*, *Fusobacteria*, and *Fusobacterium* with the incidence of ischemic and hemorrhagic strokes suggest their potential role in promoting inflammation within the gut microbiota.

Significantly higher levels of *Coprococcus* and *Roseburia hominis* within the *Firmicutes phylum* were observed in the healthy control group compared to the other two groups. Butyrate-producing bacteria, namely *Faecalibacterium*, *Roseburia*, *Coprococcus*, and *Eubacterium*, were identified in a previous study^17^. The production of butyric acid by these bacteria plays a crucial role in regulating glucose and lipid metabolism^18^, along with inhibiting the secretion of inflammatory factors and reducing infection rates^19,20^

A decrease in the abundance of butyrate-producing bacteria, specifically *Roseburia* and *Coprococcus*, in patients with ischemic or hemorrhagic stroke may lead to a reduction in glucose and lipid metabolism. This reduction, in turn, could contribute to an increase in the secretion of inflammatory factors. The intricate relationship between the gut microbiota composition, particularly the balance of butyrate-producing bacteria, and metabolic and inflammatory processes highlights the potential implications for stroke patients.

In this study, alterations in the genus composition associated with ischemic stroke primarily manifested in *Proteobacteria*, *Bacteroidetes*, and *Firmicutes*. The ischemic stroke group exhibited a notably higher abundance of Escherichia within the *Proteobacteria phylum* compared to the healthy control group. This observation aligns with findings from a prior investigation^13^, highlighting the consistent nature of *Escherichia's* elevated presence in various diseases, including inflammatory enteritis and malignant tumors^21–23^.

The *Bacteroidetes* genus *Alloprevotella* and the *Firmicutes* genus *Stomatobaculum*, both originating from the oral cavity, exhibited significant increases, particularly in diseases such as depression and anxiety^24,25^ and among the smoking population^26^. *Ruminococcus*, with the highest abundance in ischemic stroke, corroborated findings from related studies^27^. The heightened abundance of *Ruminococcus* following an ischemic stroke was associated with increased cortisol levels, upregulated expression of 5-hydroxytryptamine, and exacerbated damage to the central nervous system^28^. Genomic studies on the intestinal microflora composition of patients with cerebral ischemia further support a substantial increase in *Ruminococcus* abundance^29,30^.

Interestingly, this phenomenon was not observed in patients with hemorrhagic stroke in our current investigation. Such discrepancies suggest the presence of a distinct mechanism for *Ruminococcus* in ischemic stroke compared to hemorrhagic stroke, emphasizing the need for further exploration into the nuanced interactions between microbial composition and stroke subtypes.

In this investigation, an elevation in the abundance of *Parabacteroide*s within the *Bacteroidetes phylum*, as well as *Acetanaerobacterium*, *Lachnoclostridium*, and *Hungatella* within the *Firmicutes phylum*, was observed in association with hemorrhagic stroke. Notably, the abundance of *Parabacteroides* has been previously linked to conditions such as depression and anxiety in mouse models, showing a significant decrease following probiotic supplementation^31,32^. *Hungatella*, identified in our study, has close associations with constipation, suggesting potential implications for gastrointestinal health in individuals with hemorrhagic stroke. *Acetanaerobacterium*, an anaerobic bacterium known for hydrogen production during glucose fermentation, was also found to be increased. *Lachnoclostridium*, associated with intestinal inflammation and multiple infections^33^, demonstrated elevated abundance in the context of hemorrhagic stroke. Collectively, the observed surge in these specific bacteria, each with its distinct pathological associations, implies a potential linkage between hemorrhagic stroke and an increase in harmful microbial species. Further investigations into the intricate dynamics of these bacteria within the context of hemorrhagic stroke are warranted to deepen our understanding of their role in the disease and explore potential avenues for targeted interventions.

The intestine, acting as an endocrine and immune organ in the human body, harbors a resident microbiota that plays a crucial role in maintaining host health. Currently, the "microbiome-gut-brain axis" concept serves as a premise for investigating the relationship between stroke and the gut microbiota^34^. It posits that the gut microbiota can interact with the central nervous system through three potential pathways: the neuroendocrine system, the immune system, and the vagus nerve^35–37^, facilitating communication between the gut microbiome and the central nervous system. Furthermore, the gut microbiota can directly or indirectly influence stroke risk factors such as hypertension, diabetes, and atherosclerosis, thereby affecting stroke onset and prognosis^38^. In this study, the gut microbiota of first-onset stroke patients within 5 days of the event was analyzed, aiming to capture the gut microbiome state as close as possible to the pre-stroke condition. Previous research has reported that various factors after stroke onset can impact the gut microbiota, with severe strokes potentially causing gut dysbiosis, which in turn can influence stroke outcomes through immune-mediated mechanisms. Intriguingly, fecal microbiota transplantation has been shown to significantly improve stroke prognosis^5^. Consequently, our study effectively elucidates the impact of the gut microbiota on ischemic and hemorrhagic stroke, as well as the similarities and differences in gut microbiome profiles between these stroke subtypes. These findings have significant implications for developing targeted preventive and therapeutic strategies based on the distinct gut microbiota characteristics observed in ischemic and hemorrhagic stroke.

The structure and function of the gut microbiota are influenced by factors such as diet and genetics^39,40^. Although our study controlled for variables like body weight, diet, and gender to some extent, the sample size was relatively small, and the study was conducted exclusively on males. Due to differences in endocrine systems and lifestyles across genders, gut microbiota profiles may vary, necessitating further research with a larger sample size. Moreover, our study did not assess stroke severity, which could potentially influence the relationship between gut microbiota composition and stroke type, severity, and prognosis. Future studies should evaluate this aspect, as well as analyze the metabolic products of the gut microbiota to uncover how the distinct microbial profiles observed in ischemic and hemorrhagic stroke may impact health through their respective metabolites.

While we controlled for certain factors, the limited sample size and focus on male participants warrant caution in generalizing our findings. Expanding the study to include participants of different genders and larger cohorts would enhance the robustness and generalizability of the results. Additionally, incorporating assessments of stroke severity and characterizing the metabolic signatures associated with the observed gut microbiota alterations could provide valuable insights into the mechanistic links between dysbiosis and stroke outcomes.

To further unravel the intricacies of the impact of these microbial variations on health, it becomes imperative to extend investigations beyond taxonomic profiling. The detection and analysis of metabolites originating from differences in intestinal microflora between ischemic and hemorrhagic strokes are deemed necessary to elucidate their specific effects. This expansion in analytical scope promises a more nuanced understanding of the mechanistic implications of microbial shifts in the context of different stroke types.

Moreover, it is noteworthy that the study's participant cohort exclusively consisted of males. Recognizing the potential influence of gender on intestinal microflora, future investigations should explicitly incorporate diverse gender representations. This would facilitate a comprehensive exploration of gender-specific nuances, enriching our understanding of the interplay between gender and the intricacies of intestinal microflora dynamics in the context of stroke.

In conclusion, this study revealed distinctive patterns in the diversity of intestinal microflora between ischemic and hemorrhagic strokes compared to the health control group. The observed significant differences in microbial diversity between the two stroke types underscore the uniqueness of their respective microbial compositions. This substantiates the notion that tailored interventions targeting specific types of stroke based on their distinct microbial signatures might hold promise as an effective treatment approach. Further research endeavors, considering the limitations addressed, are essential to refine these insights and pave the way for more targeted therapeutic strategies.

## Data Availability

SRA accession number for the raw sequencing data: SRP360047: PRJNA807091, https://trace.ncbi.nlm.nih.gov/Traces/?view=study&acc=SRP360047.
